# 31 Social Deprivation, Antibiotic Prescribing Quality, and Outcomes in an Elderly Emergency Department Cohort

**DOI:** 10.1017/ash.2026.10456

**Published:** 2026-06-23

**Authors:** Mary Lou Manning, Dennis Hand, Meghan Gannon, Julia Kay, Kim McLaughlin

**Affiliations:** 1 Thomas Jefferson University; 2 Thomas Jefferson University, College of Nursing

## Abstract

**Background:** Substance use disorders (SUDs) disproportionately affect pregnant and parenting persons in the United States. Long term residential programs (RTPs) provide comprehensive, structured care that improves pregnancy and neonatal outcomes. However, shared living environments that include young children increase vulnerability to respiratory virus transmission, and evidence to guide infection prevention and control (IPC) in RTPs is limited. In December 2024, an RTP in the northeastern United States housing 13 parenting persons with 15 children experienced an outbreak of respiratory syncytial virus among children, resulting in 14 symptomatic cases, 7 laboratory-confirmed infections, and 5 hospitalizations. This study aimed to identify barriers to and enablers of IPC practices among RTP residents and staff to inform interventions tailored to this unique setting. **Methods:** In November 2025, we conducted six focus groups at the RTP: three with residents and three with staff. Due to Institutional Review Board restrictions, sessions were not audio-recorded; instead, a trained scribe captured detailed field notes. Data were analyzed using conventional thematic analysis, guided by the Capability, Opportunity, Motivation, and Behavior (COM-B) model. **Results:** Thirteen of 14 residents and 18 of 25 staff participated. Three overarching themes were identified. First, IPC capability was fragmented. Both residents and staff recognized the role of IPC in reducing respiratory virus transmission. However, residents primarily relied on social media and internet sources for information, while staff relied on guidance from healthcare professionals. Misunderstanding and absence of standardized, facility-level IPC policies led to confusion regarding appropriate cleaning/disinfecting practices and adherence to isolation/quarantine criteria. Second, IPC opportunity was constrained by contextual and structural barriers. Residents entered the program from diverse living situations, resulting in divergent interpretations of IPC norms, including hand hygiene and environmental cleaning. Shared spaces, reliance on resident-performed cleaning without consistent supervision or accountability, lack of commercial cleaning services, and inconsistent enforcement of cleaning and isolation procedures limited effective IPC implementation. Third, IPC motivations were misaligned. Staff viewed IPC practices as opportunities for life-skills development, whereas residents often perceived cleaning and isolation measures as stigmatizing or punitive, negatively affecting symptom disclosure and IPC practice adherence. **Conclusions:** Although IPC practices in RTPs aim to prevent illness among residents and children, implementation is hindered by knowledge gaps, structural constraints, perceived stigma, and misaligned motivations. These findings underscore the need for clear, standardized policies and trauma-informed, public health–focused IPC strategies. IPC professional societies are well positioned to develop context-specific guidance for this understudied residential setting.

